# Supplementary material for: Kinetic, Isotherm, and Equilibrium Investigation of Cr(VI) Ion Adsorption on Amine-Functionalized Porous Silica Beads
Source: Polymers (Basel). 2022 May 21;14(10):2104. doi: 10.3390/polym14102104 (PMC9146620; doi:10.3390/polym14102104)
Supplement: Supplementary file 1 [file polymers-14-02104-s001.zip › polymers-1723674-supplementary.pdf]

Supplementary Materials

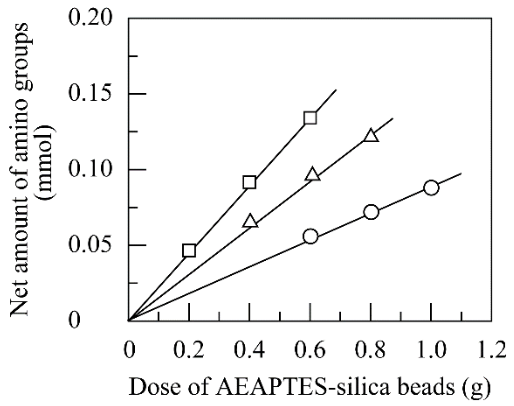

Figure S1: Changes in the net amount of amino groups with the dose of silica beads for AEAPTES-silica beads with the amount of amino groups of 0.030 (△), 0.080 (□), and 0.220 (◇) mmol/g.

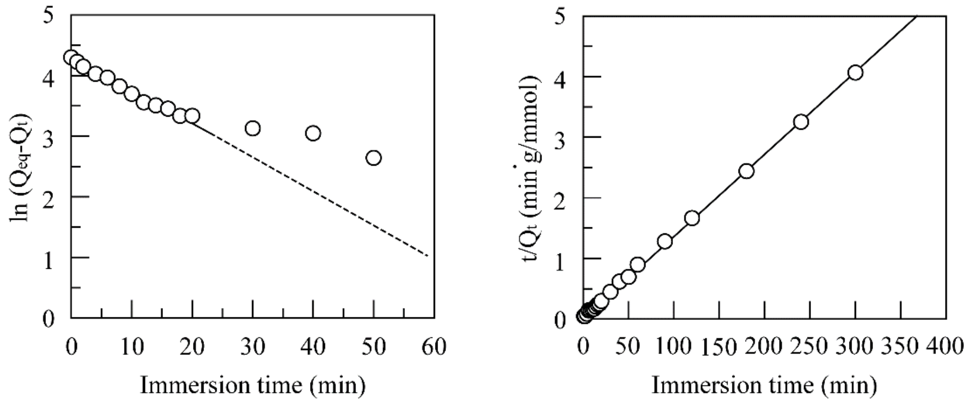

Figure S2: Determination of (a) the pseudo-first order constant, k_1, and (b) pseudo-second order constant, k_2, for adsorption of Cr(VI) ions on AEAPTES-silica beads with the amount of amino groups of 0.220 mmol/g in a 0.20 mM $K_2Cr_2O_7$ solution at pH 3.0 and 30°C.

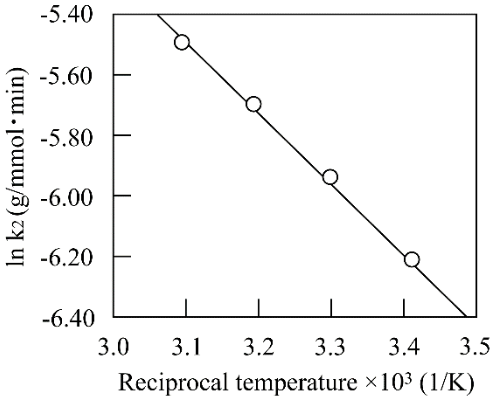

Figure S3: The linear relationship between the k_2 value and the reciprocal temperature in Arrhenius plot for adsorption of Cr(VI) ions on AEAPTES-silica beads with the amount of amino groups of 0.220 mmol/g in a 0.20 mM $K_2Cr_2O_7$ solution at pH 3.0 and 30°C.

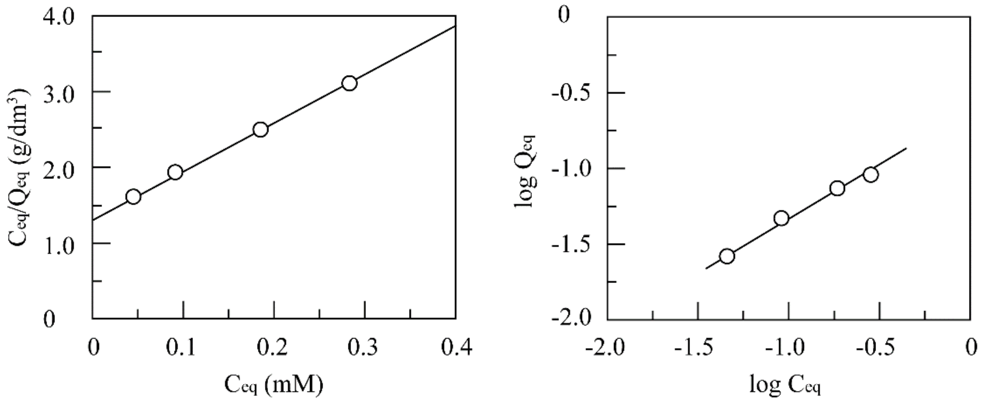

Figure S4: The linear forms of (a) Langmuir and (b) Freundlich isotherms for adsorption of Cr(VI) ions on AEAPTES-silica beads with the amount of amino groups of 0.220 mmol/g in a 0.20 mM $K_2Cr_2O_7$ solution at pH 3.0 and 30°C.
